# Radiomic Texture Analysis Mapping Predicts Areas of True Functional MRI Activity

**DOI:** 10.1038/srep25295

**Published:** 2016-05-06

**Authors:** Islam Hassan, Aikaterini Kotrotsou, Ali Shojaee Bakhtiari, Ginu A. Thomas, Jeffrey S. Weinberg, Ashok J. Kumar, Raymond Sawaya, Markus M. Luedi, Pascal O. Zinn, Rivka R. Colen

**Affiliations:** 1 Department of Diagnostic Radiology, The University of Texas MD Anderson Cancer Center, Houston, Texas, USA; 2 Department of Neurosurgery, The University of Texas MD Anderson Cancer Center, Houston, Texas, USA; 3 Department of Neurosurgery, Baylor College of Medicine, Houston, Texas, USA; 4 Department of Cancer Systems Imaging, The University of Texas MD Anderson Cancer Center, Houston, Texas, USA

## Abstract

Individual analysis of functional Magnetic Resonance Imaging (fMRI) scans requires user-adjustment of the statistical threshold in order to maximize true functional activity and eliminate false positives. In this study, we propose a novel technique that uses radiomic texture analysis (TA) features associated with heterogeneity to predict areas of true functional activity. Scans of 15 right-handed healthy volunteers were analyzed using SPM8. The resulting functional maps were thresholded to optimize visualization of language areas, resulting in 116 regions of interests (ROIs). A board-certified neuroradiologist classified different ROIs into Expected (E) and Non-Expected (NE) based on their anatomical locations. TA was performed using the mean Echo-Planner Imaging (EPI) volume, and 20 rotation-invariant texture features were obtained for each ROI. Using forward stepwise logistic regression, we built a predictive model that discriminated between E and NE areas of functional activity, with a cross-validation AUC and success rate of 79.84% and 80.19% respectively (specificity/sensitivity of 78.34%/82.61%). This study found that radiomic TA of fMRI scans may allow for determination of areas of true functional activity, and thus eliminate clinician bias.

Blood oxygen level dependent Magnetic Resonance Imaging (BOLD-MRI) is one of the most important tools in presurgical neuroimaging as it reflects the integrated synaptic activity of neurons^1^,^2^. Since the development of functional MRI (fMRI) as a technique for brain mapping, it has been extensively used in multiple clinical and research applications^3^. This can be attributed to its non-invasive nature and high spatial and temporal resolution, which allows covering of the entire brain within a short period of time^4^. Early on, fMRI was used as a tool in neurocognitive research using group analysis rather than individual analysis^5^. In group analysis, data from different subjects are averaged in order to cancel-out random contributions and increase signal-to-noise ratio (SNR)^5^. However, individual analysis is the only option in clinical decision-making such as in presurgical brain mapping.

Currently, individual analysis of fMRI data consists of steps that reduce the SNR and increase the contrast-to-noise ratio (CNR)^6^. The ultimate goal is to maximize detection of true activity and eliminate any false positives, which is achieved through adjusting the statistical threshold of fMRI map^7^. In individual analysis, determination of this threshold is arbitrary and differs from one subject to another, depending on the experience of the reporting radiologist^8^. Usually, the threshold is set to the point where maximum noise can be eliminated without affecting true activity^9^. However, it remains unclear whether the final fMRI map is a true representation of brain activity. Further, no method is known that eliminates non-essential or untrue activity that survives the arbitrary threshold and the limits of thresholding are not identified. Finally, it has to be proven if a stringent threshold always results in preservation of truly active areas.

Those questions become of extreme importance in cases of clinical applications, specifically presurgical mapping, where accuracy is pivotal for clinical decision making^10^,^11^. Despite multiple validation studies of fMRI, individual fMRI results per-se cannot be considered 100% accurate due to several factors^12^. First, fMRI is an indirect measurement of brain activity, thus it is an overstretch to assume that BOLD-signal represents activity of a specific brain region associated with the evaluated function^4^,^8^. Second, no statistical method can provide quantitative parameters to differentiate primary activity from secondary activity^7^. Third, depending only on voxel intensity to determine activity discards a lot of information that can be obtained from raw data. These aforementioned limitations highlight and beckon the need for a robust adjunct analysis that can help increase accuracy and reliability of fMRI. Such adjunct analysis must provide a quantitative platform that can capture minor differences and fluctuations of the signal intensity occurring within eloquent cortex during activity. Although quantification of functional brain activity is challenging, yet it can provide a strong predictive platform that can utilize minor computational differences between the behavior of true essential fMRI activity and other areas of functional activity captured by fMRI studies. Subsequently, radiomics analysis of medical images can play a role in detection of such minor changes that occur within the region of interest (ROI) of activity itself.

Radiomics can address this issue through mining high throughput quantitative imaging features that hold predictive power^13^. Quantitative imaging features obtained through radiomic texture analysis (TA) provide information that cannot be visually assessed^14^,^15^. The extracted features represent values from the voxel itself as well as its interrelations with the surrounding voxels. Lately, TA has acquired much attention due to the improvements in both the resolution of medical images as well as in the capabilities of computers^16^. Texture features obtained by the medical images have been correlated with disease-related changes occurring in the molecular environment, suggesting that TA can be used as a compliment of macrostructural information commonly used by the radiologists in order to better characterize pathological diseases^17^.

Ultimately, radiomic analysis can be transferred to the field of functional brain imaging, as the potential variability in behavior of different ROIs of activity can becaptured and quantified using radiomics texture features. We hypothesized that the inter-voxel changes quantified via TA can be used to predict true activity in fMRI.

The purpose of this feasibility study is to develop an automated robust method using TA to accurately predict areas of true fMRI activity. Aiming to extend the use of TA from structural images to functional images, we propose this new application of TA.

## Results

### Characteristics of fMRI activity maps

**Figure 1:**
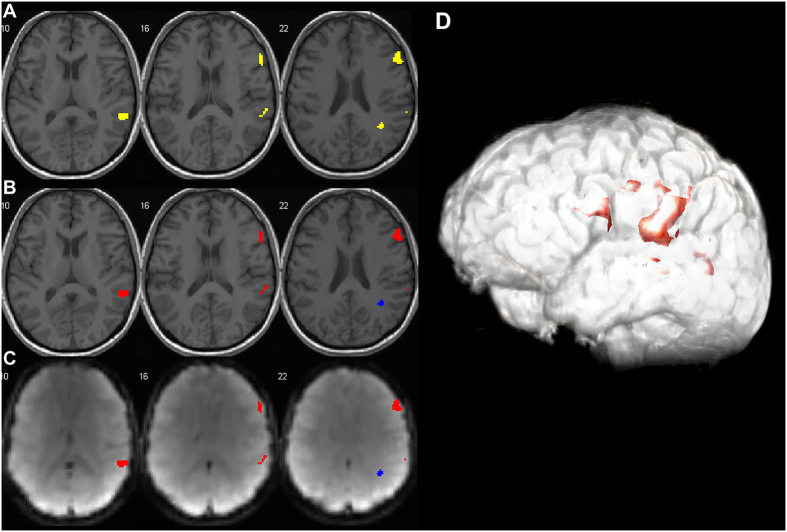
(**A**) fMRI activity maps overlaid on 3D-T1 Spoiled Gradient Echo (SPGR) to delineate anatomy and show exact location of brain activity. (**B**) fMRI activity areas classified into Expected (E) and Non-Expected (NE) based on their anatomical locations and reports from Direct Cortical Stimulation (DCS) data in literature. Blue areas represents non-expected fMRI activity within subgyral white matter, while red areas are within the expected activity area of language eloquent cortex. (**C**) fMRI activity maps overlaid on raw Echo-Plannar Imaging data. (**D**) 3D view of fMRI activity map.

A total of 116 ROIs were obtained from 15 patients’ scans; 43 ROIs were categorized as Expected (E) while the remaining 73 ROIs were categorized as Non-Expected (NE). (E/NE prevalence: 37%/63%). *P*-values of thresholded functional map ranged from 10^−3^ to 10^−12^ with a minimum cluster size of 5. Both E and NE ROIs survived the same statistical threshold. All participants showed a mixture of E and NE activity on their functional maps (Fig. 1).

### Radiomic texture feature can predict areas of true functional activity

Table 1 shows the mean, standard deviation and *p*-values for all 20 rotation-invariant features across E and NE ROIs. From the univariate analysis we can infer that texture features such as sum average and sum variance (*p*-value < 0.05) highlight significant differences between E and NE ROIs.

#### Identifying significant features

**Figure 2:**
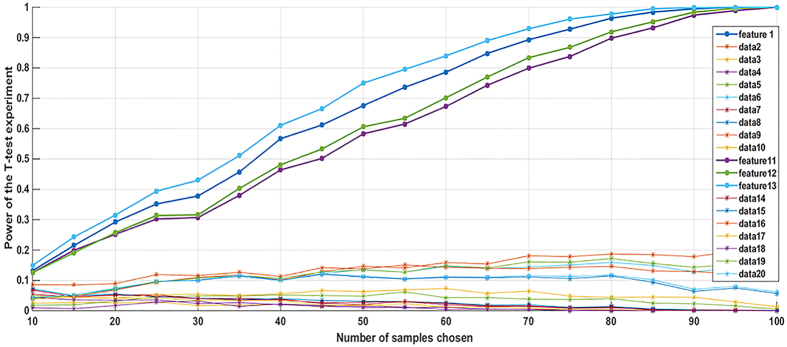
Statistical power of the student t-test for different sample sizes chosen from the feature set.

The 20 rotation invariant features were used to discriminate E from NE ROIs using logistic regression analysis. Four features showed strong linear discriminating power for the E versus NE; autocorrelation, sum average, sum variance and sum of square variance (Table 2). Figure 2 depicts the statistical power of the student t-test for different sample sizes chosen from the feature set. It is evident that for sample sizes larger than 100, the statistical power of the test is effectively 100%.

To build a robust predictive model, the feature subset chosen should contain uncorrelated features to each other. According to the correlation table (Table 3) the four significant features are strongly correlated with each other, thus we decided to skip the prior feature selection step and use stepwise logistic regression to identify the features best discriminating between E and NE ROIs.

#### Application of forward stepwise logistic regression in discriminating E from NE ROIs

To ensure cross-validation blindness towards features, we allowed the linear model decide the features it would choose without supervision. This step resulted in the inclusion of independently insignificant features inside the linear model. In order to make sure that the included features were not chosen in random in the next step we proceeded with cross-validating the model based on the features picked by the model itself. The model parameters are brought in table 4. The features that appeared in the model were dissimilarity (*p* = 0.02), Entropy (*p* = 0.002), sum variance (*p* = 0.000019) and difference entropy (*p* = 0.0002). Since the *p*-values of the individual features included in the model are all less than the threshold (0.05) the odds ratio for each feature inside the model is significantly different from 1.

**Figure 3:**
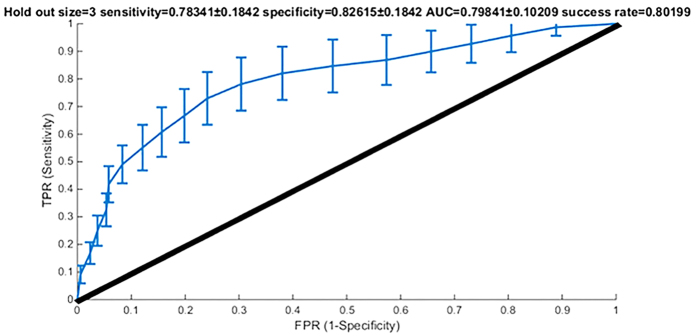
ROC plot of the logistic regression model.

In the cross-validation step, we randomly chose 12 patients to extract the ROIs for training, while we kept the rest for testing the model. In order to compensate for the statistical fluctuations we ran the cross validation 100 times. The overall success rate of the model was 80.19%, with an area under the curve (AUC) of the receiver operating characteristic (ROC) curve was 79.84%. The mean optimal specificity and sensitivity of the model was 78.34% and 82.61% respectively (Fig. 3).

## Discussion

In this study, we demonstrated the power of radiomic TA in discriminating areas of true fMRI activity from non-task-related (non-essential) and false positive activity. Our results suggest that radiomic TA can detect true functional activity with high accuracy, sensitivity and specificity. As demonstrated by cross-validated statistical analysis, our method allowed us to discriminate between E and NE ROIs with a success rate of 80.19% and 78.34% specificity and 82.61% sensitivity.

This study addresses one of the major problems in current fMRI analysis, namely the relatively low positive predictive value^8^. Although fMRI is the modality of choice for non-invasively detecting areas of functional activity relative to other brain mapping tools^8^, it is not currently possible to predict if a specific area of activity is a true area of activity without invasive measurements (i.e. direct cortical stimulation (DCS))^18^. This has limited the potential applicability of fMRI as a brain mapping tool for presurgical examinations. The need for a complementary tool that can reliably predict the nature of functional ROIs becomes imperative.

Although we depended in our classification of fMRI activity on the standardized MNI anatomical location and the experience of neuroradiologist in confirming the location of activity, yet this classification was not anecdotal; the classification of the activity depended on a meta-analysis of published data that reported incidence of speech deficit when stimulation of such location was performed intra-operatively.

Moreover, radiomics have been extensively used in the field of oncology and applied to biomedical images to reflect the underlying tumorogenesis^13^,^19^,^20^,^21^. However, extending the umbrella of radiomics to the field of fMRI reveals huge potential to achieve the goal of precision medicine^20^. Each functional imaging dataset has a large number of data that can be mined and analyzed extensively using the radiomics approach to extract an exponential amount of information and integrate it in the analysis of fMRI. Similar TA approach has been used to capture tumor heterogeneity in different forms of cancer with remarkable success^19^,^21^. Thus, it is justifiable to extrapolate the same methods to a functional application that benefit from the radiomic TA abilities to capture image and functional heterogeneity. In our study, we highlighted the potential role of radiomic TA in unveiling the true signature of cognition specifically language processing, underscoring the possible role of radiomic analysis in capturing physiological events.

TA measures the heterogeneity of image intensities in a specific region^22^. Consequently, processes that lead to changes in the distribution of the MRI signal intensity would affect the extracted features^22^,^23^. Extending this idea to fMRI, we postulated and show that TA can detect and highlight differences resulting from functional activity in the region, and quantify them into parameters that can be further used to predict the nature of activity and distinguish true activity. our univariate analysis results showed that the texture features that were found to be significant for determination of E and NE activity were sum of squares: variance (*p* = 0.004) and sum variance (*p* = 0.001) (measure of how spread the gray levels of voxel pairs are), sum average (*p* = 0.003) (measure of overall image brightness), and autocorrelation (*p* = 0.002) (measure of how voxel pairs are correlated). Mean values of the aforementioned features were higher in NE ROIs, indicating increased homogeneity compared to areas of true functional activity that was more heterogeneous (Table 1). This was further confirmed with our multivariate analysis, which illustrated robust differences in levels of textures between NE and E, with a predilection of areas of true activity exhibiting heterogeneous textures. This can be attributed to the fluctuation in voxel intensity between active and control phases of the task based fMRI^12^,^24^. Thus, it can be anticipated that since those ROIs are truly activated, there should be larger heterogeneity in the signal of the mean EPI volume.

In order that we develop a robust post-processing approach that would complement current fMRI analysis, all fMRI investigations and preprocessing techniques followed standardized, previously published protocols^25^,^26^. To avoid any bias in the quality of the fMRI, all healthy volunteers were scanned on clinical scanners and the imaging parameters used followed clinical protocols. TA was performed after the completion of the preprocessing of the fMRI; thus the methodology proposed in this work does not interfere with the preprocessing steps typically used in fMRI experiments and its translation into the clinic can be anticipated to enter with relative ease.

The present study has several limitations. Being a preliminary feasibility study, the number of healthy volunteers was relatively small; despite that sufficient statistical sample power was reached, we are currently recruiting more participants in order to determine broad scalability. Furthermore, although we performed TA using only 8 gray levels, we were able to discriminate between E and NE with high accuracy. In future studies, we aim to include a larger number of healthy participants and comprehensively investigate the effect of different number of gray levels in the predictive power of our classifier. While no validation was done using other direct brain stimulation methods, our focus was to perform a feasibility study using healthy participants, which naturally limits our ability to perform any invasive validation procedure. Our primary classification was based on previously published data using gold standard DCS^27^ and the experience of the neuroradiologist. Our goal is to extend the application of the proposed method in tumor patients as well as patients with neurocognitive and neurodegenerative diseases. This will further allow us to validate our findings by comparing them with the results from DCS. Also, inclusion of patients will allow integration of other omics data to radiomics TA. Such integration will be of paramount impact; this will make possible correlations to underlying molecular events with different patterns of cognitive stimulation, fMRI activation as well as cognitive impairment. We will also evaluate the use of multiple tasks in combination with the same methodology.

In conclusion, radiomic TA mapping of raw fMRI images can be a useful tool for detection of areas of true fMRI activity. In this study, we show the ability of TA to predict areas of true fMRI activity; additionally, TA of fMRI images can discriminate between expected and non-expected areas of activity with a success rate of 80.19%. Further, we demonstrate that the steps for TA do not interfere with the preprocessing steps of fMRI analysis. To our knowledge this is the first study that applies TA on fMRI images and highlights the potential of this technique in the field of functional neuroimaging.

## Methods

### Participants

This HIPAA-compliant study was approved by the Institutional Review Board of The University of Texas MD Anderson Cancer Center, and all participants provided written informed consent prior to inclusion into the study. All methods were performed in accordance with the approved guidelines. Prospective participants were contacted via email notification sent to volunteers who had subscribed to our hospital study volunteer server list. After detailed information was provided, a telephone screening was performed for the following inclusion criteria: age above 18 years, no prior brain surgeries or neuropsychological disorders and no contraindications to MRI. To exclude potential confounding effects that foreign language has on functional activity^22^, only native English speakers were considered for inclusion. Final analyses was based on 15 right-handed healthy volunteers (6 males, 9 females) aged between 22 and 66 years, with mean age 38.2 years. This pilot study was initiated on February 2014 and concluded on June 2015.

### MRI Acquisition

Functional and structural images were acquired using a General Electric 3.0 Tesla Discovery MR750 MR scanner (GE Healthcare, Waukesha, WI, USA) with a 32-channel birdcage head coil and high order shim. Each participant was presented with the language tasks on a backlit projection screen. Functional images were acquired using a T2^*^-weighted BOLD sequence (repetition time (TR) = 2000 msec, echo time (TE) = 30 msec, matrix size = 64 × 64, field-of-view (FOV) = 24 × 24 cm, slice thickness = 4 mm with no intersection gap). This slice prescription allowed full coverage of the brain. A high-resolution 3D Spoiled Gradient Echo (SPGR) T1-weighted (T1WI) image was acquired for anatomic reference (TR/TE = 6 msec/2 msec, matrix size = 256 × 256, FOV = 24 × 24 cm, slice thickness = 1.2 mm with no intersection gap).

### Task Paradigm

Participants performed 3 different language tasks including word generation, category naming and sentence completion as previously described^26^,^28^. For the purpose of this study, only the sentence completion paradigm was further analyzed, as this task has shown reliable activity in the language area^11^. All participants were trained on the task prior to scanning using a PowerPoint presentation. A block-design experiment was used due to its high sensitivity in identifying differences between control and active conditions^29^.

In the block-designed experiment, during active condition, the participant was presented with an incomplete sentence and asked to complete it, for example (“Astronauts uses rockets to go to outer ____”). This was followed by a control condition, during which the participant was presented with scrambled letters arranged in the form of nonsense words to create a gibberish sentence, for example (“Xbg rhc hgxgr Jknrhc sp ______”). The active and control conditions were matched in sentence length. The task consisted of alternating 6 active and 6 control conditions (total 12 conditions); each condition had a duration of 20 sec and 10 iterative whole brain volumes were acquired during that time resulting in a total number of 120 iterative whole brain volumes.

### Image analysis

**Figure 4:**
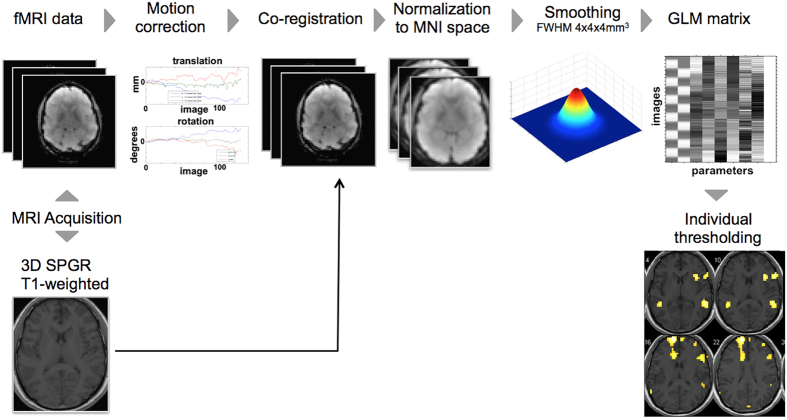
Diagrammatic illustration of the fMRI analysis processing pipeline.

fMRI image analysis was done using statistical parametric mapping 8 (SPM8) software (Wellcome Department of Cognitive Neurology, London, UK) (Fig. 4). The images were obtained in DICOM format and then transformed to an analyzed format for SPM8 analysis. First, the volumes were motion corrected by registering each volume to the first acquired slice using affine registration (12 degrees-of-freedom). The resultant motion-corrected volumes were co-registered to the anatomical T1WI. Subsequently, volumes were normalized to the Montreal Neurological Institute (MNI) template. This step provided a reference atlas and facilitated the identification of areas of true functional activity. A Gaussian filter with 4 × 4 × 4 mm^3^ full-width-at-half-maximum (FWHM) was applied to smooth the data, thus increase the signal to noise ratio^30^. Then, this data was used to generate a functional map that represents the changes in signal intensity occurring during task stimulation using the general linear model design (GLM) matrix^11^. The map was individually thresholded to show maximum activity at language areas with uncorrected *p*-values (ranging from 10^−3^–10^−12^)^31^.

### Identification of areas of true functional activity versus false positives

When results from Direct Cortical Stimulation (DCS) are not available, identification of true functional activity is performed by evaluating the anatomical location of each ROI of the functional map along with literature findings using DCS. The anatomical location of each ROI of the functional map was identified using the result of the normalization step to the MNI space. This was simply performed using xjView software (http://www.alivelearn.net/xjview) that provides a summary report of the number of voxels of each ROI belonging to each anatomical area. In the next step, the individually thresholded functional maps along with the anatomical results obtained through xjView were reviewed by a board certified neuroradiologist (R.R.C., 7 years of experience in fMRI) who evaluated and confirmed the anatomical location of activity and eventually classified activity based on the anatomical gyral location of fMRI activity into expected (E) and non-expected (NE) activity^27^. When a cluster of activity was located partially in an E ROI and partially in a NE ROI, we used xjView software (http://www.alivelearn.net/xjview) cluster report feature to determine the number of voxels per each ROI and the maximum intensity, the cluster was eventually labelled according to the higher Z-score. Activity in cortical regions related to language such as Brodmann’s 44, 45 and 22 as well as inferior and middle frontal gyri, and superior temporal gyrus were classified as E regions. Areas of non-task-related activity such as cerebellum, sub-gyral white matter, insula, post-central gyrus, supra-marginal gyrus and superior frontal gyrus were classified as NE regions. Additionally, 2 ROIs per participant were drawn in areas of no functional activity on EPI using Mango Software (Research Imaging Institute, University of Texas Health Science Center at San Antonio, TX, USA; http://ric.uthscsa.edu/mango/) and labeled as NE. Size and location of the aforementioned ROI of inactivity was fixed across different subjects; this was done to establish a ground truth of a true NE region.

### Radiomic Texture Analysis

Radiomic features were computed using the mean volume of the normalized non-smoothed data. TA was performed using our in-house software created on MatLab environment (Mathworks, Natick, MA). For the analysis, we used 2-dimensional gray level co-occurrence matrices (GLCMs) for each ROI as proposed by Haralick in 1973^22^. A 2-dimensional GLCM is a lattice (nxn) matrix, where n is the number of gray levels that contains the joint probability of two adjacent pixels in a given angular direction Θ. In the case of 2-dimensional GLCM, we applied 4 possible angular directions corresponding to Θ = 0, 45, 90, 135 degrees.

Prior to texture feature calculation, we performed a normalization process to reduce the number of gray levels in order to prevent sparseness in the GLCMs^32^. For the purpose of this study 8 gray levels were used, thus the initial range of gray levels as defined by the voxel intensities was divided into 8 equal gbins and the values in each bin were assigned a single gray level. We calculated 20 radiomic texture features for each angular direction and subsequently averaged to produce a rotation invariant measure of each feature^22^,^32^,^33^. A summary of the calculated features is presented in Table 5.

### Statistical Analysis

The final radiomic texture matrix was 116 × 20 (116 ROIs, 20 rotation invariant features). To find the relationship between texture features and the E versus NE status of ROIs, We applied the forward stepwise multivariate logistic regression model. We considered only linear relationships between the covariates and the logit of regression function. The inclusion and exclusion criteria for the stepwise regression were *p*-values less than 0.05 and greater than 0.15, respectively. In each step, the feature satisfying the inclusion criteria with the lowest *p*-value was included and concordantly the feature satisfying the exclusion criteria with the highest *p*-value was excluded from the model. We used the Akaike’s information criterion (AIC) to avoid over-fitting. In the training step, we analyzed our model based on the ROIs explicitly extracted from a training set of the patients (93 ROIs (80%)), and we subsequently tested it on the mutually exclusive set of patients (23 ROIs (20%)). By extracting the training and test sets from different patients, we assured that our model is not biased by the intrinsic feature character of individual patients. Statistical analysis was performed using software (Matlab R2014b, MathWorks, Natick, Mass) (SPSS, version 22; SPSS statistics, Armonk, NY).

## Additional Information

**How to cite this article**: Hassan, I. *et al.* Radiomic Texture Analysis Mapping Predicts Areas of True Functional MRI Activity. *Sci. Rep.* **6**, 25295; doi: 10.1038/srep25295 (2016).

## Figures and Tables

**Table 1:** Descriptive statistics for the expected and non-expected ROIs.

Texture feature	Expected (E)		Non-Expected (NE)		T test *P*-Value
	Mean	StandardDeviation	Mean	StandardDeviation	
Autocorrelation*	27.5	6.9	33.4	10.8	0.002
Contrast	0.3	0.2	0.2	0.4	0.288
Correlation	0.6	0.3	0.6	0.3	0.123
Cluster Prominence	8.0	10.7	9.1	20.8	0.757
Cluster Shade	−1.1	2.0	−0.7	2.3	0.320
Dissimilarity	0.3	0.2	0.2	0.2	0.116
Energy	0.5	0.3	0.5	0.3	0.627
Entropy	0.5	0.3	0.4	0.3	0.529
Homogeneity	0.9	0.1	0.9	0.1	0.076
Maximum probability	0.6	0.2	0.6	0.2	0.719
Sum of squares: variance*	27.9	7.3	33.4	10.8	0.004
Sum average*	10.4	1.3	11.4	1.9	0.003
Sum variance*	101.5	25.4	124.5	39.8	0.001
Sum entropy	0.4	0.2	0.4	0.2	0.588
Difference variance	0.3	0.2	0.2	0.4	0.288
Difference entropy	0.2	0.1	0.2	0.1	0.109
Information measure of correlation1	−0.3	0.2	−0.4	0.2	0.232
Information measure of correlation2	0.4	0.2	0.4	0.2	0.911
Inverse difference normalized (INN)	1.0	0.0	1.0	0.0	0.101
Inverse difference moment normalized	1.0	0.0	1.0	0.0	0.261

The *P*-value is obtained using Student’s t-test for the equality of means.

^*^indicates significant difference (P < 0.05).

**Table 2:** Descriptive statistics of prominent texture features obtained using logistic regression analysis.

Feature name	*P*-value
Autocorrelation	0.002
Sum average	0.003
Sum variance	0.001
Sum of squares: variance	0.004

**Table 3:** The correlation between the 4 significant features.

	Autocorrelation	Sum of squares: variance	Sum average	Sum variance
**Autocorrelation**	1.00	0.99	0.99	0.99
**Sum of squares: variance**	0.99	1.00	0.99	0.99
**Sum average**	0.99	0.99	1.00	0.99
**Sum variance**	0.99	0.99	0.99	1.00

**Table 4:** Logistic regression table parameters.

Feature	Estimate	t-State	*P*-Value
**(Intercept)**	0.007	0.005	0.995
**Dissimilarity**	10.319	2.239	0.025
**Entropy**	11.220	3.081060216	0.002
**Sum variance**	0.005	4.269	1.96 × 10^−5^
**Difference entropy**	−60.877	3.720	0.0002

**Table 5:** Texture Features used in the Analysis.

Gray Level Co-occurrence Matrix–Haralick^22^	Energy, Contrast, Correlation, Variance, Sum average, Sum variance, Sum entropy, Entropy, Difference variance, Difference Entropy, Information measure of Correlation 1, Information measure of correlation 2
Gray Level Co-occurrence Matrix–Soh^33^	Autocorrelation, Cluster Prominence, Cluster Shade, Dissimilarity, Homogeneity, Maximum Probability
Gray Level Co-occurrence Matrix–Clausi^32^	Inverse difference normalized, Inverse difference moment normalized
